# Bevacizumab Dose Affects the Severity of Adverse Events in Gynecologic Malignancies

**DOI:** 10.3389/fphar.2019.00426

**Published:** 2019-04-26

**Authors:** Shu-Ping Lee, Heng-Cheng Hsu, Yi-Jou Tai, Yu-Li Chen, Ying-Cheng Chiang, Chi-An Chen, Wen-Fang Cheng

**Affiliations:** ^1^Department of Obstetrics and Gynecology, College of Medicine, National Taiwan University, Taipei, Taiwan; ^2^Department of Obstetrics and Gynecology, National Taiwan University Hospital Yun-Lin Branch, Douliou, Taiwan; ^3^Graduate Institute of Clinical Medicine, College of Medicine, National Taiwan University, Taipei, Taiwan; ^4^Department of Obstetrics and Gynecology, National Taiwan University Hospital Hsin-Chu Branch, Hsinchu, Taiwan; ^5^Graduate Institute of Oncology, College of Medicine, National Taiwan University, Taipei, Taiwan

**Keywords:** ovarian cancer, bevacizumab, adverse events, hypertension, proteinuria

## Abstract

In this retrospective study, we investigated adverse events and outcomes in patients treated with bevacizumab for ovarian, fallopian tube, or primary peritoneal cancers at a single hospital. We determined the cumulative incidences of various bevacizumab-related adverse events and the correlation between dose and adverse event incidences. We analyzed data from 154 patients that received 251 rounds of bevacizumab as first-line, first salvage, >2 salvage treatments. Adverse events of any grade were observed in 121 (78.6%) patients; at least one grade 3 or 4 adverse event occurred in 32 (20.8%) patients. The two most common events were proteinuria (38.3%) and hypertension (33.8%). The first-line treatment group displayed significantly higher frequencies of hypertension (52.7% vs. 18.9% vs. 15.5%, *p* < 0.001), wound complications (9.1% vs. 0% vs. 1.2%, *p* = 0.010), arthralgia (29.1% vs. 11.3% vs. 8.3%, *p* = 0.003), and reduced range of joint motion (14.5% vs. 5.7% vs. 3.6%, *p* = 0.046), compared to those in the first and >2 lines salvage groups, respectively (Kruskal–Wallis test). The cumulative incidences of all grades and grades 3/4 of hypertension cumulative incidence plateaued at around 30% for all grades and 10% for grades 3 and 4, at bevacizumab doses above 8080 and 3510 mg, respectively. The proteinuria cumulative incidence plateaued at around 35% for all grades and 3% for grades 3 and 4, at bevacizumab doses above 11,190 and 4530 mg, respectively. We concluded that, in this realistic clinical population, different kinds and higher cumulative incidences of adverse events were observed compared to those reported in previous clinical trials. Moreover, bevacizumab doses showed cumulative toxicity and plateau effects on hypertension and proteinuria.

## Introduction

In recent years, ovarian cancer has become the most lethal of gynecologic malignancies. In 2018, an estimated 22,240 and 1,100 new cases of ovarian cancer in the United States (Siegel et al., 2018) and Taiwan (Chang et al., 2018). The lack of characteristic symptoms and effective screening tests for early detection of ovarian cancer has led to delayed diagnoses and poor prognoses (Hamilton et al., 2009). Most patients are diagnosed at an advanced stage, with an overall five-year survival of less than 40% (Colombo et al., 2006; Hamilton et al., 2009). Ovarian cancer treatments include surgical staging with maximal cytoreduction and systemic treatments, mainly chemotherapy (Colombo et al., 2014). Systemic treatments for ovarian cancer have changed little over the past 15 years. The standard first-line chemotherapy is a carboplatin/paclitaxel combination. Systemic chemotherapy has improved progression-free survival (PFS) and overall survival (OS) in women with advanced-stage ovarian cancer (McGuire et al., 1996). However, despite the high initial response rate to standard chemotherapy, the majority of patients with ovarian cancer eventually relapses and develops resistance to multiple chemotherapeutic agents (Colombo et al., 2014).

Various attempts had been made to improve systemic treatments for ovarian cancer. In addition to chemotherapeutic agents, molecular-targeting agents have been developed with improvements in our understanding of tumor biology (Diab and Muallem, 2017). Among these molecular-targeting agents, the two most promising are anti-angiogenic agents and poly-ADP ribose polymerase inhibitors (Burger et al., 2011; Perren et al., 2011; Ledermann et al., 2012; Coleman et al., 2017; Lin and Kraus, 2017; Swisher et al., 2017; Komiyama et al., 2018). Bevacizumab is a monoclonal antibody that targets vascular endothelial growth factor (VEGF). It inhibits angiogenesis, tumor growth, and metastases, and it has been approved for the treatment of many kinds of human malignancies including ovarian cancer (National Cancer Institute, 2018). Multiple clinical trials have demonstrated that bevacizumab had promising effects in the treatment of advanced, metastatic, or recurrent ovarian cancer (Burger et al., 2011; Perren et al., 2011; Aghajanian et al., 2012; Pujade-Lauraine et al., 2014; Coleman et al., 2017; Komiyama et al., 2018). Two phase 3 trials (GOG-218 and ICON 7) showed that bevacizumab maintenance therapy showed beneficial effects in the treatment of newly diagnosed advanced-stage ovarian cancer (Burger et al., 2011; Perren et al., 2011). Two other phase 3 trials (OCEANS and GOG-213) led to the approval of bevacizumab for treating platinum-sensitive ovarian cancer (Aghajanian et al., 2012; Coleman et al., 2017), and one phase 3 trial (AURELIA) led to the approval of bevacizumab for treating platinum-resistant ovarian cancer (Pujade-Lauraine et al., 2014). The effectiveness of chemotherapy with bevacizumab had also been investigated in platinum-resistant recurrent ovarian cancer patients, which showed consistency with previous clinical trials (Lee et al., 2019). Combination of bevacizumab with other targeted agents also showed promising response in treating recurrent ovarian cancer patients (Monk et al., 2016). However, increased toxicity while combining with everolimus was found in a phase 2 study (Tew et al., 2018).

Many adverse events associated with bevacizumab were observed during clinical trials, including hypertension, proteinuria, gastrointestinal events, wound complications, and thromboembolism (Burger et al., 2011; Perren et al., 2011; Aghajanian et al., 2012; Pujade-Lauraine et al., 2014; Coleman et al., 2017). However, in most of those clinical trials, patients were highly selected with standardized treatment protocols. Consequently, the experiences and data from clinical trials are not fully applicable to clinical practice, because many patients in the real world do not meet the inclusion criteria of clinical trials, and the real-world regimen may be personalized. Therefore, the present retrospective study aimed to evaluate the cumulative incidence of bevacizumab-related adverse events in an unselected group of patients with ovarian cancer. The results of this study might provide clinicians with more realistic clinical information when prescribing bevacizumab for patients with ovarian cancer in clinical practice.

## Patients and Methods

### Patient Population

From August 2009 to December 2017, 154 women with ovarian, fallopian tube, or primary peritoneal cancers that had received bevacizumab in one medical center were retrospectively identified from the institutional records. Bevacizumab can be given in combination with chemotherapy or alone as a single agent. We included women that received bevacizumab in three different scenarios: as a front-line adjuvant chemotherapy after surgery, as first salvage chemotherapy, or as >2 lines of salvage chemotherapy after relapse. In some patients, bevacizumab was first administered with chemotherapy, then continued as a maintenance therapy after chemotherapy was completed. This study protocol was approved by the Institutional Review Board of the hospital.

### Data Collection

Demographic and clinical data were retrieved from medical records in the hospital’s centralized database. These data included the age at diagnosis, International Federation of Obstetrics and Gynecologic (FIGO) stage, tumor histology and grade, type of surgery, and types and cycles of chemotherapy. Disease staging was based on the 2014 FIGO staging system. Optimal debulking surgery was defined as residual tumors with maximal diameters less than 1 cm; the others were defined as suboptimal debulking surgery. Disease recurrence was defined as elevated tumor marker levels (greater than twice the upper limit of normal); abnormal radiological findings (including computerized tomography, magnetic resonance imaging, and positron emission tomography); or histological proof from biopsy analyses. Patients were defined as chemo-sensitive, when they experienced disease recurrence later than 6 months after completing the front-line adjuvant chemotherapy. Patients were defined as chemo-resistant, when they experienced disease recurrence within 6 months after, or disease progression during front-line adjuvant chemotherapy.

We calculated the dose and duration of bevacizumab administration. For calculations on the dose of bevacizumab, patients that received only one shot of bevacizumab were excluded. Adverse events potentially associated with bevacizumab were described according to the Common Terminology Criteria for Adverse Events, version 4.0. The adverse events of special interest in this study included hypertension, proteinuria, respiratory tract hemorrhage (including epistaxis), gastrointestinal hemorrhage, thromboembolic events, wound complications, gastrointestinal perforation, arthralgia, reduced range of joint motion, and musculoskeletal pain. Patients were separated into three groups, based on the lines of previous chemotherapies: zero (55 who received a front-line adjuvant treatment), one (53 who received a first salvage treatment), and (84 who received two or more lines of salvage treatment). One patient could receive more than one round of bevacizumab in adjuvant and/or salvage treatments. We calculated the number of adverse events and the dose of bevacizumab taken before each recorded adverse event. Some patients could have experienced more than one relapse and could have received more than one round of bevacizumab during different relapses; therefore, we calculated the initial and cumulative doses. The initial dose was defined as the bevacizumab dose given at the beginning of a new round of bevacizumab, before the recorded adverse event occurred. The cumulative dose was defined as the lifetime total dose of bevacizumab, from the first time prescription of bevacizumab until the recorded adverse event occurred.

#### Follow-Up and Analysis

Overall survival was calculated as the time interval from the date of bevacizumab introduction to the date of death from any cause. PFS was defined as the time interval from the date of bevacizumab introduction to clinically defined recurrence, disease progression, or death from any cause, whichever occurred first. Statistical analyses were carried out with the Statistical Package of Social Studies (SPSS) version 22 (SPSS Inc., Chicago, IL) for Windows. Comparisons between unpaired groups were performed with the Mann–Whitney *U* test and the Kruskal–Wallis *H* test. The bevacizumab doses were assessed as continuous variables and analyzed with the Mann–Whitney *U* test and the Kruskal–Wallis *H* test. Survival curves were generated with the Kaplan–Meier method, and differences in survival curves were calculated with the log rank test. A *p*-value less than 0.05 was considered statistically significant.

## Results

### Clinico-Pathologic Characteristics of the 154 Gynecologic Cancer Patients Treated With Bevacizumab

Among the 154 patients included, 132 (85.7%) had ovarian cancer, 16 (10.4%) had primary peritoneal cancer, and 6 (3.9%) had fallopian tube cancer (Table 1). The median age at diagnosis was 54 years (range 27–82 years). The median follow-up time was 36 months (range 4–221 months). The majority of patients presented with advanced-stage diseases (stage III or IV) (79.2%). The most common histological cancer type was serous (63.0%), followed by clear cell (20.8%), and endometrioid (6.5%). High grade tumors (grades 2 and 3) comprised 94.2% of all tumors. Eighty-five patients (55.2%) underwent optimal debulking surgery, and 145 patients (94.1%) underwent platinum-based adjuvant chemotherapy (90.9% combined with paclitaxel and 3.2% combined with cyclophosphamide). One hundred and thirty-six patients (88.3%) experienced disease recurrence during follow-up. Among patients with recurrences, the most common prior medical history was hypertension (21.4%), followed by diabetes mellitus (6.5%). Fifty-six (36.4%) patients received more than one round of bevacizumab during different disease statuses. Therefore, the data included 251 rounds of bevacizumab that were prescribed for 154 patients. Fifty-five (35.7%) patients received bevacizumab in front-line adjuvant treatment, and 116 (75.3%) in salvage treatment (Table 2).

**TABLE 1:** Clinico-pathologic characteristics of 154 women of malignancies treated with bevacizumab.

Characteristics	Patient number (%)
Type of gynecologic cancer	
Ovarian cancer	132 (85.7%)
Tubal cancer	6 (3.9%)
Primary peritoneal cancer	16 (10.4%)
FIGO stage	
I	20 (13.0%)
II	12 (7.8%)
III	82 (53.2%)
IV	40 (26.0%)
Histologic type	
Serous	97 (63.0%)
Clear cell	32 (20.8%)
Endometrioid	10 (6.5%)
Mucinous	3 (1.9%)
Carcinosarcoma	6 (3.9%)
Carcinoma	4 (2.6%)
Mixed type^a^	2 (1.3%)
Histological grade	
Low (Grade 1)	5 (3.2%)
High (Grade 2 and 3)	145 (94.2%)
Not specified	4 (2.6%)
Type of surgery	
Optimal	85 (55.2%)
Suboptimal	69 (44.8%)
Adjuvant chemotherapy	
Platinum + Paclitaxel	140 (90.9%)
Platinum + Cyclophosphamide	5 (3.2%)
Others^b^	2 (1.3%)
Not done	7 (4.5%)
Chemo-response	
No recurrence^c^	18 (11.7%)
Chemo-sensitive	69 (44.8%)
Chemo-resistant	67 (43.5%)
Prior medical history	
Hypertension	33 (21.4%)
Diabetes mellitus	10 (6.5%)
Coronary artery disease	2 (1.3%)
Gastrointestinal ulcer	25 (16.2%)

*^a^One with endometrioid, clear cell, and serous types, one with endometrioid and serous types; ^b^One treated with cisplatin and doxorubicin, one treated with paclitaxel alone; ^c^Follow up for at least 3 months.*

**TABLE 2:** Characteristics of 154 gynecologic malignancies patients prescribing 251 rounds of bevacizumab.

Characteristics	Patient number (%)
Front-line adjuvant therapy	55 (35.7%)
Recurrent treatment	116 (75.3%)
1st line salvage therapy^a^	53 (34.4%)
>2nd line salvage therapy^a^	84 (54.5%)
Chemotherapeutic regimen(s) combined with	Rounds^a^ (%)
bevacizumab	
Platinum + Paclitaxel	105 (41.8%)
Paclitaxel	17 (6.8%)
Platinum	7 (2.8%)
Lipodox + Platinum	55 (21.9%)
Topotecan + Platinum	14 (5.6%)
Gemcitabine + Platinum + Paclitaxel	22 (8.8%)
Etoposide + Platinum	3 (1.2%)
Others^b^	11 (4.4%)
Bevacizumab alone	17 (6.8%)
Maintenance therapy with Bevacizumab	
Yes	68 (27.1%)
No	183 (72.9%)
**Dose of bevacizumab, mg/kg/week^c^**	
<1.5	68 (27.1%)
1.5–1.99	74 (29.5%)
2–3	58 (23.1%)
>3	19 (7.6%)
**Ascites**	
No	105 (41.8%)
Yes	146 (58.2%)
**Pleural effusion**	
No	209 (83.3%)
Yes	42 (16.7%)
**Gastrointestinal obstruction**	
No	236 (94.0%)
Yes	15 (6.0%)

*^a^One patient may have more than one rounds of bevacizumab, ^b^One with cisplatin and ifosfamide, one with ifosfamide, one with docetaxel, one with FOLFOX (oxaliplatin + 5-FU), one with gemcitabine, one with gemcitabine and 5-FU, two with gemcitabine and paclitaxel, one with oxaliplatin and docetaxel, three with cisplatin and high dose 5-FU, ^c^32 rounds were excluded from calculation because only one dose of bevacizumab was given.*

At the time of prescribing bevacizumab, the patients had been administered a median of two lines of chemotherapy (range, 0–13). Eighty-four (54.5%) patients had received at least two lines of chemotherapy; 61 patients (39.6%) received at least three lines of chemotherapy; and 13 patients (8.4%) received at least five lines of chemotherapy. In the front-line adjuvant treatment group, the median duration of using bevacizumab (including combination therapies and maintenance therapies) was 278 days (range 21–1152 days). In the recurrent treatment group, the median duration of using bevacizumab was 69 days (range 14–1260 days).

The majority (93.2%) of bevacizumab rounds were administered with chemotherapy. The most common chemotherapeutic regimen was platinum combined with paclitaxel (41.8%). Sixty-eight rounds (27.1%) of bevacizumab treatment were continued as maintenance therapy. The median duration of maintenance therapy was 146 days (range 14–1113 days). Various bevacizumab dosing regimens were adopted. The dose of 2–3 mg/kg/week was administered in fifty-eight rounds (23.1%) of bevacizumab. In seventy-four (29.5%) and sixty-eight (27.1%) rounds, the doses of bevacizumab were 1.5–1.99 mg/kg/week and less than 1.5 mg/kg/week, respectively.

### The Majority of Bevacizumab-Related Adverse Events Was Mild During Both Front-Line and Salvage Treatments

Among the 154 study patients, 121 (78.6%) experienced at least one adverse event of any grade. One hundred and thirteen patients (73.4%) experienced at least one adverse event of grade 1 or 2, and 32 patients (20.8%) experienced at least one adverse event of grade 3 or 4. No grade 5 adverse event was encountered. The adverse events potentially associated with bevacizumab treatment are summarized in Table 3. The two most common adverse events were proteinuria (38.3%) and hypertension (33.8%). Two patients treated with bevacizumab had gastrointestinal perforation or leakage. Of these, the first patient underwent a right hemicolectomy with secondary debulking, followed by salvage chemotherapy. A bowel perforation occurred 4 months after surgery. The patient received conservative treatment, but died of disease 3 months later. The second patient received a right hemicolectomy in primary debulking surgery. Gastrointestinal leakage over the surgical anastomosis site was suspected after 2 cycles of adjuvant chemotherapy, due to free-air within the right subdiaphramatic area. Supportive care was provided, and chemotherapy was reintroduced 10 days later, after the leakage spontaneously healed. Bevacizumab combined with adjuvant chemotherapy was prescribed since next cycle of treatment.

**TABLE 3:** Adverse events during bevacizumab treatment in 154 gynecologic malignancies women.

Category	Patient number (%)^a^			
Grade	Grade 1–2	Grade 3–4	Grade 5	Total
Hypertension	30 (19.5%)	22 (14.3%)	0	52 (33.8%)
Proteinuria	49 (31.8%)	10 (6.5%)	0	59 (38.3%)
Gastrointestinal hemorrhage	28 (18.2%)	1 (0.6%)	0	29 (18.8%)
Respiratory tract hemorrhage	24 (15.6%)	1 (0.6%)	0	25 (16.2%)
Thrombo-embolic event	10 (6.5%)	3 (1.9%)	0	13 (8.4%)
Wound complication	4 (2.6%)	2 (1.3%)	0	6 (3.9%)
Gastrointestinal perforation	2 (1.3%)	0 (0%)	0	2 (1.3%)
Arthralgia	28 (18.2%)	1 (0.6%)	0	29 (18.8%)
Range of joint motion decrease	3 (8.4%)	1 (0.6%)	0	14 (9.1%)
Musculoskeletal pain	48 (31.2%)	0 (0%)	0	48 (31.2%)

*^a^One patient may have more than one above-mentioned adverse event, so the total percentage was more than 100%.*

### The Cumulative Incidence of Bevacizumab-Related Adverse Events Was Higher With Front-Line Treatment Than With Salvage Treatment

We compared bevacizumab-related adverse events that occurred in the front-line adjuvant treatment group and the two salvage (1st and >2nd) treatment groups. As shown in Table 4, some adverse events were more common in patients in the front-line adjuvant treatment group. For example, hypertension was observed in a higher percentage of patients in the front-line adjuvant treatment group between these three groups (52.7% vs. 18.9% vs. 15.5%, *p* < 0.001, Kruskal–Wallis test). Additionally, the front-line adjuvant treatment group had significantly higher cumulative incidences of wound complications (9.1% vs. 0% vs. 1.2%, *p* = 0.010), arthralgia (29.1% vs. 11.3% vs. 8.3%, *p* = 0.003), and reduced range of joint motion (14.5% vs. 5.7% vs. 3.6%, *p* = 0.046; all assessed with the Kruskal–Wallis test) in all the three groups. The cumulative incidences of other adverse events, including proteinuria, gastrointestinal hemorrhage, respiratory tract hemorrhage, thromboembolic event, and gastrointestinal perforation, were not significantly different among these three groups.

**TABLE 4:** Chemotherapeutic line and the development of adverse events during bevacizumab treatment in 154 gynecologic malignancies women.

Line(s) of chemotherapeutic regimens	Front-line	1st salvage	>2nd salvage	*p*^b^
**Patient number (%)^a^**	55	53	84	
**Adverse events**				
Hypertension	29 (52.7%)	10 (18.9%)	13 (15.5%)	<0.001
Proteinuria	15 (27.3%)	13 (24.5%)	31 (36.9%)	0.25
Gastrointestinal hemorrhage	5 (9.1%)	7 (13.2%)	17 (20.2%)	0.18
Respiratory tract hemorrhage	5 (9.1%)	7 (13.2%)	13 (15.5%)	0.55
Thrombo-embolic event	1 (1.8%)	5 (9.4%)	7 (8.3%)	0.22
Wound complication	5 (9.1%)	0 (0%)	1 (1.2%)	0.010
Gastrointestinal perforation	1 (1.8%)	0 (0%)	1 (1.2%)	0.64
Arthralgia	16 (29.1%)	6 (11.3%)	7 (8.3%)	0.003
Range of joint motion decrease	8 (14.5%)	3 (5.7%)	3 (3.6%)	0.046
Musculoskeletal pain	14 (25.5%)	11 (20.8%)	23 (27.4%)	0.68

*^a^One patient may have more than one above-mentioned adverse event, so the total percentage was more than 100%; ^b^*p*-value by Kruskal–Wallis *H* test.*

### Bevacizumab-Related Adverse Events Were Induced by Different Initial, but Not Cumulative Doses in Front-Line and Salvage Treatment Groups

We calculated the initial and cumulative bevacizumab doses administered to patients with ovarian cancer that displayed therapy-related adverse events (Table 5). The median initial dose required to induce proteinuria was significantly higher in the front-line adjuvant treatment group than in the two salvage groups (5100 vs. 600 and 600 mg, respectively; *p* < 0.001, Kruskal–Wallis *H* test). The front-line adjuvant treatment group also displayed significantly higher median initial doses for inducing arthralgia (2700 vs. 1000 and 400 mg, *p* = 0.007) and reduced range of joint motion (7223 vs. 600 and 1200 mg, *p* = 0.041) compared to the salvage treatment groups (both Kruskal–Wallis *H* test). The median initial doses for inducing the other bevacizumab-related adverse events were not different between these three groups. Moreover, there was no significant difference in the cumulative doses required to induce bevacizumab-related adverse events in all three groups (Table 5).

**TABLE 5:** Previous chemotherapeutic line(s) and dose of bevacizumab in developing adverse events in 154 gynecologic malignancies women.

Line(s) of chemotherapeutic regimens		Front- line	1st salvage	>2nd salvage	

Median dose (mg)					*p*
Hypertension	I	1700	2100	1300	$0.27{}^{a}$
	C	1700	2100	2000	$0.87{}^{a}$
Proteinuria	I	5100	600	600	$<0.001{}^{a}$
	C	5100	1500	2500	$0.071{}^{a}$
Gastrointestinal hemorrhage	I	2700	600	600	$0.34{}^{a}$
	C	2700	1500	1200	$0.83{}^{a}$
Respiratory tract hemorrhage	I	800	900	800	$0.77{}^{a}$
	C	800	900	1500	$0.18{}^{a}$
Thrombo-embolic events	I	3300	600	600	$0.27{}^{a}$
	C	3300	600	1200	$0.40{}^{a}$
Wound complication	I	500	–	400	$0.55{}^{b}$
	C	500	–	400	$0.55{}^{b}$
Gastrointestinal perforation	I	600	–	300	$0.32{}^{b}$
	C	600	–	2100	$0.32{}^{b}$
Arthralgia	I	2700	1000	400	$0.007{}^{a}$
	C	2700	2100	900	$0.11{}^{a}$
Range of joint motion decrease	I	7223	600	1200	$0.041{}^{a}$
	C	7223	2700	1200	$0.15{}^{a}$
Musculoskeletal pain	I	1400	1200	800	$0.14{}^{a}$
	C	1400	1200	900	$0.79{}^{a}$

*I: Initial dose, C: cumulative dose, ^a^by Kruskal–Wallis *H* test, ^b^by Mann–Whitney *U* test.*

These results indicated that the cumulative incidences of some adverse events were higher only with initial doses, not with cumulative doses. Our results indicated that bevacizumab had cumulative toxic effects on some adverse events, such as proteinuria and arthralgia.

### Cumulative Incidences of Bevacizumab-Related Adverse Events With Cumulative Bevacizumab Doses

**FIGURE 1:**
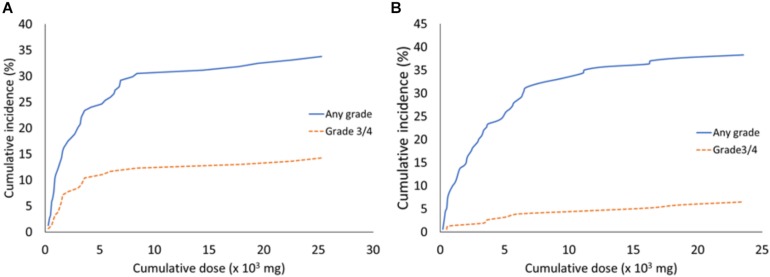
The cumulative incidences of hypertension and proteinuria associated with different median cumulative bevacizumab doses. **(A)** All grades and grades 3/4 of hypertension that developed with the indicated bevacizumab doses. **(B)** All grades and grades 3/4 of proteinuria that developed with the indicated bevacizumab doses.

We next investigated whether the cumulative incidences of bevacizumab-related adverse events, such as hypertension or proteinuria, increased with the increasing cumulative bevacizumab doses. We evaluated the cumulative incidences of all grades of hypertension at different median cumulative bevacizumab doses (Figure 1A). We found that 5% of patients developed hypertension at 567 mg bevacizumab, 10% at 885 mg, 15% at 1555 mg, and the cumulative incidences of hypertension plateaued at around 30% above the dose of 8080 mg. In addition, the cumulative incidences of grades 3 and 4 hypertension were 5% at 1370 mg, 10% at 3510 mg, and the cumulative incidences plateaued above 3510 mg (Figure 1A).

The cumulative incidences of all grades of proteinuria are shown with the median cumulative doses of bevacizumab in Figure 1B. We found that 5% of patients developed proteinuria at 470 mg bevacizumab, 10% at 960 mg, 15% at 2005 mg, and 35% at 11,190 mg. The cumulative incidences of proteinuria plateaued at around 35% above the dose of 11,190 mg. The cumulative incidences of grades 3 and 4 proteinuria were 3% at 4530 mg, and they plateaued above this dose (Figure 1B).

#### Progression-Free Survival and Overall Survival in Patients Treated With Bevacizumab

**FIGURE 2:**
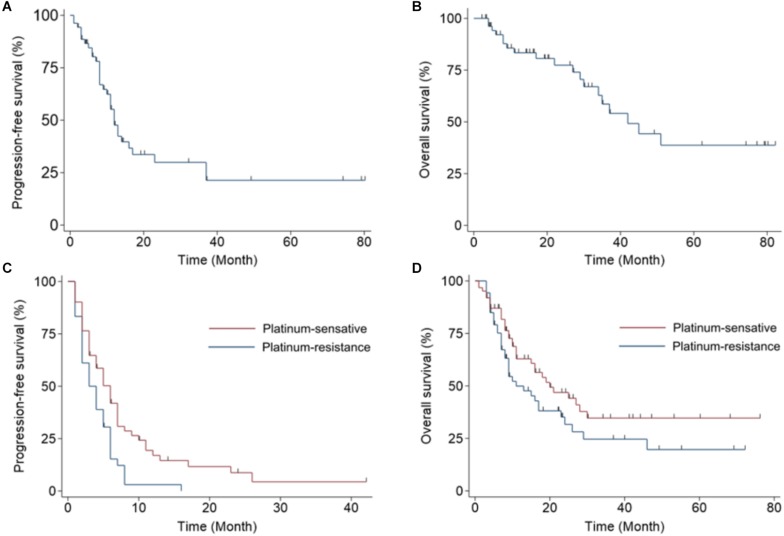
Kaplan–Meier plots display progression-free survival (PFS) and overall survival (OS) in women with ovarian cancer treated with bevacizumab. **(A)** PFS and **(B)** OS for patients that received bevacizumab as a front-line adjuvant treatment. **(C)** PFS and **(D)** OS for patients that received bevacizumab as a salvage treatment; patients are separated into a chemo-sensitive group and a chemo-resistant group.

We next evaluated the PFS and OS of patients treated with bevacizumab. Patients in the front-line adjuvant treatment group had a median PFS of 10 months (range, 1–80 months; Figure 2A) and a median OS of 19 months (range, 2–82 months; Figure 2B). Patients in the salvage treatment groups had median 4 months (range, 1–42 months) of PFS (Figure 2C) and 11 months (range, 1–76 months) of OS, respectively, in the chemo-sensitive group. And 2 months (range, 1–16 months) of PFS (Figure 2C) and 9 months (range, 3–72 months) of OS (Figure 2D) were observed in the chemo-resistant group.

## Discussion

Angiogenesis plays an important role in cancer progression and metastasis, including ovarian cancer (Gavalas et al., 2013). The mechanism of angiogenesis is highly complicated, involving multiple molecular pathways. VEGF is one of the key elements in angiogenic pathways, which mediates vessel permeability, promote endothelial cell activation, proliferation, migration, and survival (Hicklin and Ellis, 2005). By binding onto VEGF and neutralizing its activity, bevacizumab inhibits tumor angiogenesis, and hence inhibits tumor growth and metastasis (Presta et al., 1997).

Adverse events related to target therapy are different from side effects related to chemotherapy. Previous studies have shown that adverse events commonly related to bevacizumab include hypertension, proteinuria, different kinds of bleeding, and thromboembolic events. The adverse events related to bevacizumab are tolerable and manageable. Only 2% of patients discontinued therapy due to an event attributable to bevacizumab in the GOG213 trial (Coleman et al., 2017). In this cohort of patients with ovarian cancer, the three most common adverse events related to bevacizumab were proteinuria (38.3%), hypertension (33.8%), and musculoskeletal pain (31.2%). This study reported events that have not been commonly reported in previous clinical trials such as arthralgia, reduced range of joint motion, and musculoskeletal pain. We found that, when bevacizumab was used in front-line chemotherapy, it was associated with higher cumulative incidences of hypertension, wound complications, and musculoskeletal adverse events, compared to when bevacizumab given in the second or third lines of chemotherapy. We also investigated the bevacizumab dosage that induced the adverse events. The initial doses of bevacizumab required to develop proteinuria, arthralgia, and reduced range of joint motion were higher for patients in the front-line therapy group compared to patients treated with bevacizumab in second and third-line therapies. In contrast, the cumulative doses required to induce adverse events were not different among the front-line, second-line, and third-line therapy groups.

Gastrointestinal perforation occurs rather infrequently, but it is life-threatening when it occurs during anti-VEGF treatment (Hurwitz and Saini, 2006). The mechanism of gastrointestinal perforation is not well understood; it is speculated to arise at sites of bowel resection, anastomosis, abscesses, or diverticula (Kabbinavar et al., 2005; Giantonio, 2006). In phase III studies on patients with ovarian cancer treated with bevacizumab, the cumulative incidences of bowel perforation ranged from 0 to 2.8% (Burger et al., 2011; Perren et al., 2011; Aghajanian et al., 2012; Pujade-Lauraine et al., 2014). In the present study, two patients had grade 3 or higher gastrointestinal perforations. Both of these patients had received a right hemicolectomy during debulking surgery. In one of the two patients, the gastrointestinal leakage healed spontaneously, and bevacizumab was re-introduced. Thus, our series revealed that the re-introduction of bevacizumab was not absolutely contraindicated for patients with gastrointestinal perforations or leakage. We recommend that patients with recent or prior bowel resections should be carefully monitored when applying bevacizumab.

In the present study, the majority of gastrointestinal perforations/leakages occurred during the early dosing of bevacizumab, rather than during maintenance or off treatment. No previous study specifically documented the possibly that a specific dose of bevacizumab might be required for the occurrence of this serious adverse event. The present study showed that gastrointestinal perforation/leakage occurred at the 600 mg bevacizumab dose in the front-line setting and at a 300 mg dose in the recurrent setting. We recommend that patients that receive intestinal resections during early bevacizumab dosing must be closely monitored, particularly when bevacizumab is combined with chemotherapy, to ensure the early detection of gastrointestinal perforations/leakages.

Angiogenesis is essential in wound healing (Bates and Jones, 2003); thus, anti-VEGF agents may interfere with wound healing. The influence of bevacizumab on wound healing in patients with colorectal cancer was previously investigated in an analysis of pooled results from phase II and III trial (Scappaticci et al., 2005). They found that bevacizumab and chemotherapy did not significantly increase the frequency of wound complications compared to chemotherapy alone, when treatments were given at least 28 days after the operation (Scappaticci et al., 2005). However, bevacizumab and chemotherapy was associated with more wound complications (*n* = 10, 13%) than chemotherapy alone (*n* = 1, 3.4%), when treatments were administered before the operation (Scappaticci et al., 2005). There were 10 wound complications that occurred with bevacizumab prior to surgery (Scappaticci et al., 2005). In the present study, the first serious wound dehiscence due to bevacizumab treatment occurred 34 days after debulking surgery. That patient had an abdominal wall tumor that was excised, and the wound was repaired with an anterolateral thigh flap. Wound debridement was performed, and 40 days later, bevacizumab was re-introduced and continued thereafter. The second patient occurred when bevacizumab was re-introduced 1 day before a port-A implantation, and the port-A developed wound dehiscence. These findings indicated that bevacizumab could influence wound healing; thus patients should be closely monitored for the possibility of wound dehiscence, when bevacizumab is given shortly after surgery.

Clinical trials for testing bevacizumab were restricted with several criteria. Two previous phase III clinical trials, OCEANS and AURELIA, only recruited patients with ovarian cancer that had undergone less than three prior chemotherapeutic lines of cytotoxic agents. This criterion was applied to prevent serious adverse effects, such as bowel perforation (Aghajanian et al., 2012; Pujade-Lauraine et al., 2014), based on results from a previous phase II study by Cannistra et al. (2007). There was 23.8 or 0% of patients with three or <3 prior chemotherapy had bowel perforation. However, in real clinical settings, patients that have undergone three or more lines of prior chemotherapy (heavy pretreatments) are potential candidates for bevacizumab therapy. A retrospective study by Martin et al. (2016) showed that bevacizumab could be safely given, even after heavy pretreatments, when physicians avoided selecting patients with tumors that showed bowel involvement. Their results revealed that only 1.6% of patients with heavy pretreatments developed bevacizumab-related bowel perforations (Martin et al., 2016). The present study included 61/154 (39.6%) patients that underwent heavy pretreatments and only one (1.6%) patient developed a bowel perforation. However, in our series, patients that had symptoms and/or signs of bowel obstruction, ileus, or a tumor with suspected bowel involvement (detected with imaging) were excluded from bevacizumab treatment. Therefore, our results suggested that the incidence of bevacizumab-related bowel perforations could be effectively reduced in patients with prior heavy pretreatments, by carefully selecting suitable candidates.

Among our patients with ovarian cancer, 31.2% displayed bevacizumab-related musculoskeletal adverse events, such as arthralgia, reduced range of joint motion, and pain. These adverse events were not frequently reported in prior bevacizumab clinical trials. Previous investigators reported that paclitaxel treatment was also associated with arthralgia and joint discomfort (Kurtz et al., 2011; Sugiyama et al., 2016). Patients with ovarian cancer received bevacizumab combined with platinum and paclitaxel in a front-line treatment more frequently than in a salvage treatment. Therefore, we hypothesized that these musculoskeletal symptoms or adverse events might not be caused by bevacizumab; instead, they might have been associated with or aggravated by the combination of paclitaxel-based chemotherapy and bevacizumab. Further investigation is needed to investigate this possibility.

Hypertension was the most common adverse event associated with bevacizumab treatment. Hypertension occurred in 12 to 41% of patients with ovarian cancer treated with bevacizumab (Burger et al., 2011; Perren et al., 2011; Aghajanian et al., 2012; Pujade-Lauraine et al., 2014; Coleman et al., 2017). The mechanism underlying hypertension development with bevacizumab seems to be related to a reduced production of nitric oxide in the walls of arterioles and resistance vessels (Horowitz et al., 1997). VEGF increases nitric oxide production, which affects vasodilatation; conversely, anti-VEGF agents reduce NO production (Hood et al., 1998). Reductions in NO levels could induce vasoconstriction, which in turn, can induce hypertension (Hermann et al., 2006). In our series, hypertension occurred more frequently in the frontline bevacizumab treatment group than in the recurrence treatment groups (52.7% vs. 18.9% vs. 15.5%, *p* < 0.001, Kruskal–Wallis *H* test). Similarly, Oza et al. (2017) reported that hypertension occurred in early bevacizumab administration. We hypothesized that the difference between our series and previous clinical trials might be due to racial differences: the Mongolian genetic background of our series might confer susceptibility to developing hypertension during bevacizumab treatment. Alternatively, because bevacizumab is a humanized monoclonal antibody, we hypothesized that it might generate antigens that induce hypertension in some patients with specific genetic backgrounds.

Proteinuria is a common adverse event associated with bevacizumab. The development of proteinuria after blocking VEGF signal transduction demonstrated that VEGF was essential for renal function (Eremina et al., 2003; Schrijvers et al., 2004). In phase III trials of bevacizumab for ovarian cancer, the incidence of grade 3 proteinuria ranged from 0.7 to 8% (Burger et al., 2011; Perren et al., 2011; Aghajanian et al., 2012; Pujade-Lauraine et al., 2014; Coleman et al., 2017). Oza et al. (2017) reported that proteinuria appeared in the early months of bevacizumab administration. To our knowledge, no previous study investigated the relationship between the bevacizumab dose and the incidence of proteinuria. In our patients, the cumulative incidence of proteinuria was similar between the frontline and recurrence groups of patients (27.3% vs. 24.5% vs. 36.9%, *p* = 0.250, Kruskal–Wallis *H* test). However, we found that the bevacizumab doses required to induce proteinuria were higher during frontline administration (5100 mg) than during recurrence (600 and 600 mg, *p* < 0.001, Kruskal–Wallis *H* test).

The incidences of bevacizumab-related adverse events correlated with the bevacizumab dose. Previously, no study had investigated the bevacizumab dosage that induced hypertension, proteinuria, and other adverse events. The present study demonstrated that adverse events were correlated with the cumulative dose of bevacizumab and identified the bevacizumab dose that induced adverse events. We found that a median dose of 1700 to 2100 mg bevacizumab induced hypertension. Moreover, we determined that the cumulative incidence of bevacizumab-induced hypertension was around 30% at doses of 8080 mg bevacizumab, and the cumulative incidence plateaued at higher doses. The cumulative incidence of proteinuria was 35% above a dose of 11,190 mg, and the cumulative incidence plateaued at higher doses. This finding suggested that bevacizumab possessed cumulative toxicity in inducing hypertension and proteinuria, but the toxicity plateaued at high doses. Therefore, we recommend that patients should be carefully monitored when doses of 8000 mg bevacizumab are given, to watch for hypertension and proteinuria development. Indeed, the total dose should be calculated before prescribing bevacizumab for patients that previously received bevacizumab treatments, and these patients should be monitored for the occurrence of adverse events.

This study had some limitations. First, it had the inherent limitations of a retrospective study, and it was conducted at only one institute. Because we based our findings on medical records, adverse events could have been under-reported. Because all patients were recruited from one institute, patient bias could not be ruled out. Moreover, all recruited patients were Mongolian. Therefore, a racial bias might have influenced the results. In addition, the decision to administer bevacizumab depended on agreement between the physician and patient, which might have caused a selection bias. However, the fact that all patients were Mongolian might also be a strength of the study. Most previous trials were conducted in western nations, which did not include many Asians or Mongolians. Therefore, our cohort might have addressed an unmet need in research. Future prospective studies, such as phase IV or post-marketing studies, will be necessary to address this question. The strengths of this study were that all patients had ovarian, fallopian tube, or primary peritoneal cancers, and they were recruited without the strict selective criteria used in phase II or III clinical trials. We even included more patients that were heavily pretreated compared to previous trials. Thus, our cohort was more representative of the real clinical situation, because as a chronic disease, ovarian cancer is continuously treated with line after line of chemotherapy.

In conclusion, this study revealed different types of adverse events and higher cumulative incidences than those reported in previous clinical trials that tested bevacizumab for treating gynecological malignancies. Moreover, we observed that bevacizumab had cumulative and plateau effects on hypertension and proteinuria events.

## Ethics Statement

This study was approved by the Research Ethics Committee at the National Taiwan University Hospital (201706023RINC). All of the patients’ data were fully anonymized before we accessed them and the Research Ethics Committee waived the requirement for informed consent.

## Author Contributions

S-PL and W-FC conceived and designed the study. S-PL, H-CH, Y-JT, Y-LC, and Y-CC performed acquisition of data. S-PL, H-CH, Y-JT, and W-FC analyzed and interpreted the data. S-PL and W-FC drafted the manuscript. C-AC critically revised the manuscript.

## Conflict of Interest Statement

The authors declare that the research was conducted in the absence of any commercial or financial relationships that could be construed as a potential conflict of interest.[extraconflict]
